# A tradition and an epidemic: determinants of the campylobacteriosis winter peak in Switzerland

**DOI:** 10.1007/s10654-014-9917-0

**Published:** 2014-07-03

**Authors:** Philipp Justus Bless, Claudia Schmutz, Kathrin Suter, Marianne Jost, Jan Hattendorf, Mirjam Mäusezahl-Feuz, Daniel Mäusezahl

**Affiliations:** 1 Swiss Tropical and Public Health Institute, Socinstrasse 57, 4002 Basel, Switzerland; 2 University of Basel, Petersplatz 1, 4003 Basel, Switzerland; 3 Federal Office of Public Health, Schwarztorstrasse 96, 3003 Bern, Switzerland

**Keywords:** *Campylobacter*, Notification system, Case–control study, Switzerland, Gastroenteritis, Food borne diseases

## Abstract

Campylobacteriosis is the most frequently reported food borne infection in Switzerland. We investigated determinants of infections and illness experience in wintertime. A case–control study was conducted in Switzerland between December 2012 and February 2013. Cases were recruited among laboratory-confirmed campylobacteriosis patients. Population-based controls were matched according to age group, sex and canton of residence. We determined risk factors associated with campylobacteriosis, and help seeking behaviour and illness perception. The multivariable analysis identified two factors associated with an increased risk for campylobacteriosis: consumption of meat fondue (matched odds ratio [mOR] 4.0, 95 % confidence interval [CI] 2.3–7.1) and travelling abroad (mOR 2.7, 95 % CI 1.1–6.4). Univariable analysis among meat fondue consumers revealed chicken as the type of meat with the highest risk of disease (mOR 3.8, 95 % CI 1.1–13.5). Most frequently reported signs and symptoms among patients were diarrhoea (98 %), abdominal pain (81 %), fever (66 %), nausea (44 %) and vomiting (34 %). The median perceived disease severity was 8 on a 1-to-10 rating scale. Patients reported a median duration of illness of 7 days and 14 % were hospitalised. Meat fondues, mostly “*Fondue chinoise*”, traditionally consumed during the festive season in Switzerland, are the major driver of the epidemic campylobacteriosis peak in wintertime. At these meals, individual handling and consumption of chicken meat may play an important role in disease transmission. Laboratory-confirmed patients are severely ill and hospitalisation rate is considerable. Public health measures such as decontamination of chicken meat and improved food handling behaviour at the individual level are urgently needed.

## Introduction

In recent years, campylobacteriosis emerged as the most commonly reported zoonosis in Europe, including Switzerland [1, 2]. In 2012, the notification rate was 106 cases per 100,000 population corresponding to 8,567 laboratory confirmed cases [3], the highest rate since campylobacteriosis became a notifiable disease in 1988 [1]. By registering only laboratory-confirmed cases, substantial underreporting is very likely.

Human *Campylobacter* infections generally lead to self-limiting, acute gastroenteritis with diarrhoea, abdominal pain, fever, vomiting and bloody stool as commonly reported symptoms [4]. Patients suffering of a severe infection and pregnant or immunocompromised patients require antibiotic treatment [5]. Rare but serious sequels of *Campylobacter* infections include reactive arthritis, febrile convulsions and Guillain–Barré syndrome [4] and contribute considerably to morbidity and economic costs of campylobacteriosis [6, 7]. Varying case-definitions, targeted age groups and co-morbidities, methodologies, and follow-up periods result in a broad range of reported case-fatality rates. Risk factors for sporadic and outbreak-related *Campylobacter* infections have been extensively studied [8, 9]. Some 50–80 % of sporadic human *Campylobacter* infections are attributable to chicken as a reservoir either through transmission via handling and consumption of poultry, eating undercooked poultry or via contact with live poultry [10–14]. Recent case–control studies identified chicken consumption as source of infection for 24–29 % of all cases [14]. Similarly, consuming chicken is an attributable risk exposure for 27 % of campylobacteriosis cases in Switzerland [15]. Indirect evidence for an association between chicken consumption and human campylobacteriosis is provided by: (1) a significant reduction of campylobacteriosis case notifications after large-scale market-withdrawals of chicken due to dioxin-contaminated feed components [16] or an avian influenza outbreak [17] and (2) congruent seasonality patterns of the incidence of campylobacteriosis in humans and *Campylobacter* colonisation of broiler flocks [18]. Other reported exposure risks originate from drinking unsafe water, consuming raw milk and unpasteurised dairy products, eating barbecued meat, travelling abroad and from contact with farm animals and pets [2, 8, 9]. Campylobacteriosis outbreaks in Europe are rare, accounting for about 2 % of campylobacteriosis cases only [14, 19]. They are mostly associated with consumption of contaminated drinking water, raw milk and chicken products [9, 19, 20].

In temperate regions, seasonal patterns of human campylobacteriosis exist with an increased incidence during summer months [21, 22]. In Switzerland and Germany, seasonal patterns exhibit two distinct peaks: one in summer and one in winter [1, 23]. Reasons for this remain speculative: in Switzerland, suspected causes for both peaks include handling of raw and consumption of undercooked meat from barbecuing and from preparing a traditional meat fondue, a festive Christmas and New Year’s dish, which implicates the handling of raw meet by the consumer at the table [1]. The objectives for this study were to investigate determinants of the campylobacteriosis winter peak in Switzerland and to elucidate illness perception, symptomatology, and help seeking patterns of campylobacteriosis patients.

## Methods

A case–control study recruiting prospectively laboratory-confirmed campylobacteriosis cases and population-based controls was conducted between December 2012 and February 2013.

The National Notification System for Infectious Diseases (NNSID) of the Swiss Federal Office of Public Health (SFOPH) covers entire Switzerland. *Campylobacter* infections must be mandatorily reported by diagnostic laboratories. Four private laboratories, covering entire Switzerland and diagnosing about one-third of all notified cases, participated in case recruitment from 21st December 2012 until 24th January 2013.

Considering the seasonal nature of *Campylobacter* infections, the study commenced after the SFOPH enacted that the mandatory notifications of participating laboratories had to include person-identifiable data as stipulated by the Swiss Epidemics Act.

### Cases

All cases reported by the four laboratories to the NNSID were screened for eligibility. Eligibility criteria for cases were age ≥5 years and Swiss residency. Cases were excluded if they reported antibiotic treatment 4 weeks prior to disease onset or were not speaking German, French or Italian.

### Controls

Controls were selected from a random sample of the Swiss population obtained from the Federal Statistical Office. They were matched for sex, age group and canton of residence. Controls were excluded if they reported a diarrhoeal illness 4 weeks prior to the corresponding case’s disease onset. In addition, the same exclusion criteria as for cases were applied.

### Sample size

The study was designed to detect an effect size [odds ratio (OR)] of 2.5, with a power of 80 % at a two-sided significance level of 0.05 assuming a case-to-control ratio of 1:1. Rejection rates were estimated at 50 % for cases and 75 % for controls. To achieve a sample size of 100 cases and 100 controls and to account for refusals and for exclusions after enrolment, sampling foresaw contacting 300 cases and 600 controls. All eligible controls were included, resulting in a case-to-control ratio ranging from 1:1 to 1:4.

### Recruitment process

Within 24 h upon receiving a positive laboratory report we sent an information letter together with a photo-illustrated questionnaire to the case by priority mail. The same package was mailed to four matched controls within 24 h after completion of the case interview. Following the written notice cases and controls were contacted by telephone and, after giving verbal consent to participate, either interviewed immediately or a suitable appointment for the interview was fixed. If controls refused participation, additional controls were selected until at least one per case could be interviewed. Cases and controls were excluded after 15 unsuccessful call attempts or if no telephone number was available in the telephone directory or upon request via postal mail. For participants <15 years, letters were sent to their parents and either parent was interviewed as surrogate.

### Questionnaire

The questionnaire comprised a section on food- and non-food exposures and, for cases, a part on illness experience. It contained questions regarding food consumption, origin of meat, eating and hygiene behaviour, contacts to animals and humans, knowledge about food borne pathogens, recent travel history, occupational exposure and co-morbidity. For both, cases and matched controls, exposure information was collected for the 7 days preceding the onset of the case’s disease, except for travel history (preceding 2 weeks). For case interviews, the questionnaire addressed morbidity, health seeking behaviour and treatment. Computer-assisted telephone interviews using LimeSurvey software were performed. In parallel, participants were encouraged to follow the interview questions in the photo-illustrated questionnaire.

### Statistical analyses

Collected data were exported to Stata 10.1 (Stata Corporation). Pair-matched analyses were performed where applicable and matched odds ratios (mOR) are presented. Univariable conditional logistic regressions were performed. Variables with cells containing zero values in contingency tables were analysed using exact logistic regression.

For the multivariable conditional logistic regression we considered variables with *p* ≤ 0.2 in the univariable analysis. In case of correlated predictor variables only the one which was biologically more plausible was kept in the model. In addition, we performed a subgroup analysis investigating risk factors among persons who reported fondue consumption.

The population attributable fraction (PAF) was calculated for each statistically significant risk factor of the multivariable model as difference of nationwide observed cases and expected cases in absence of the risk factor. Expected cases were calculated using the multivariable mOR, frequency of exposure among cases and controls and the sex-, age- and canton-specific prevalence of *Campylobacter* notifications during the study period.

Subsequent exploratory data analysis including additional subgroup and stratified analyses was conducted in order to assist in the interpretation and to generate new hypotheses. When conditional analysis was not possible the results are presented descriptively.

## Results

### Response rate and basic characteristics of study participants

**Fig. 1:**
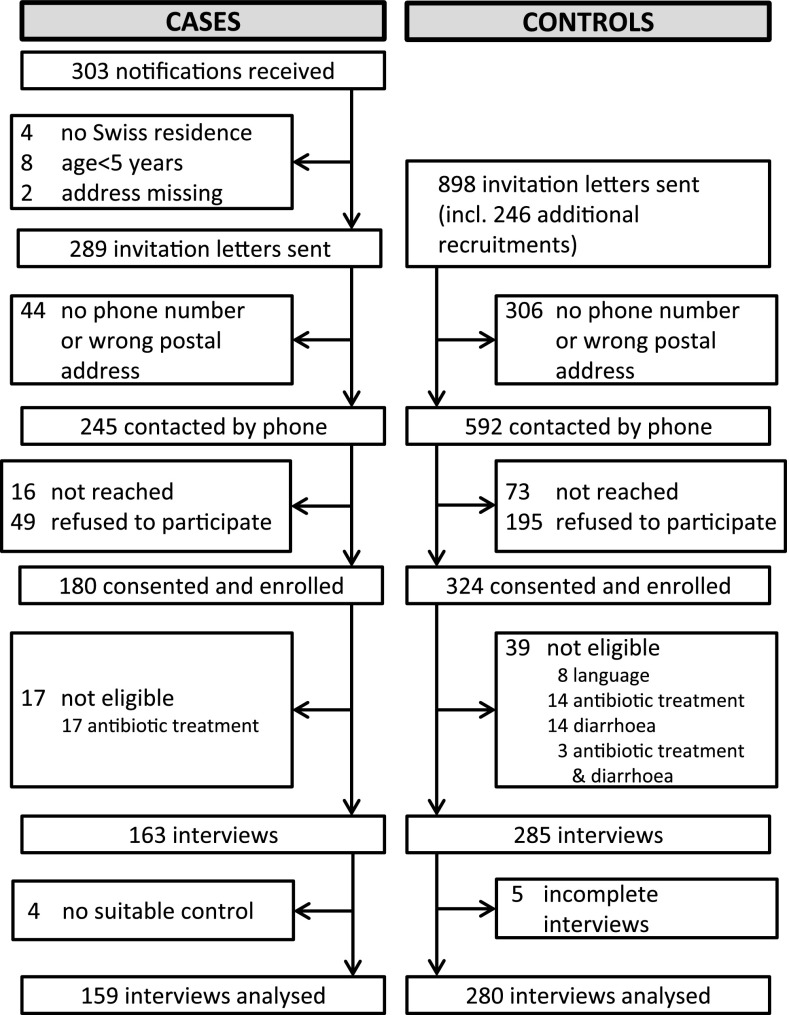
Study profile of participants enrolled and recruited in the case–control study on *Campylobacter* infections in Switzerland, December 2012–February 2013

A total of 303 campylobacteriosis case notifications were received by the study team. After exclusion of cases <5 years and non-Swiss residency, 289 cases and 898 controls were invited to participate in the study (Fig. 1). We enrolled 180 (62 %) cases and 324 (36 %) controls of which 159 (55 %) cases and 280 (31 %) controls were included in the analysis. Case-to-control matching ratios were 1:1 for 72, 1:2 for 57, 1:3 for 26 and 1:4 for 4 cases, respectively. Participating cases represented 15 % of all registered laboratory-confirmed campylobacteriosis cases during the study period.

The median number of call attempts was 2 for cases and 3 for controls. The median time period for cases between disease onset and interview was 15 days (range 5–63 days). Median age of participants was 38 years and the sex ratio was close to unity. Both study groups were consistent with regard to most socio-demographic characteristics (Table 1). An imbalance was observed in nationality as only 8 (5.0 %) cases compared to 40 (14.3 %) controls were not Swiss nationals.

**Table 1:** Socio-demographic characteristics of 159 cases and 280 controls who participated in the case–control study on campylobacteriosis in Switzerland, December 2012–February 2013

Characteristic	Cases, n (%)	Controls, n (%)
*Sex*		
Male	82 (51.6)	143 (51.1)
Female	77 (48.4)	137 (48.9)
*Age groups (years)*		
5–9	10 (6.3)	20 (7.1)
10–14	6 (3.8)	8 (2.9)
15–19	11 (6.9)	18 (6.4)
20–24	18 (11.3)	39 (13.9)
25–29	15 (9.4)	24 (8.6)
30–44	39 (24.5)	65 (23.2)
45–59	36 (22.6)	61 (21.8)
60–74	16 (10.1)	31 (11.1)
75+	8 (5.0)	14 (5.0)
*Nationality*		
Swiss	151 (95.0)	240 (85.7)
Foreign	8 (5.0)	40 (14.3)
*Education* ^a^		
Low education	109 (68.6)	173 (61.8)
High education	50 (31.4)	107 (38.2)

^a^Low education implies none, compulsory and vocational education. High education implies high school degree, university degree or other higher education

### Risk factors for campylobacteriosis during the festive season

#### Univariable conditional logistic regression analysis

**Fig. 2:**
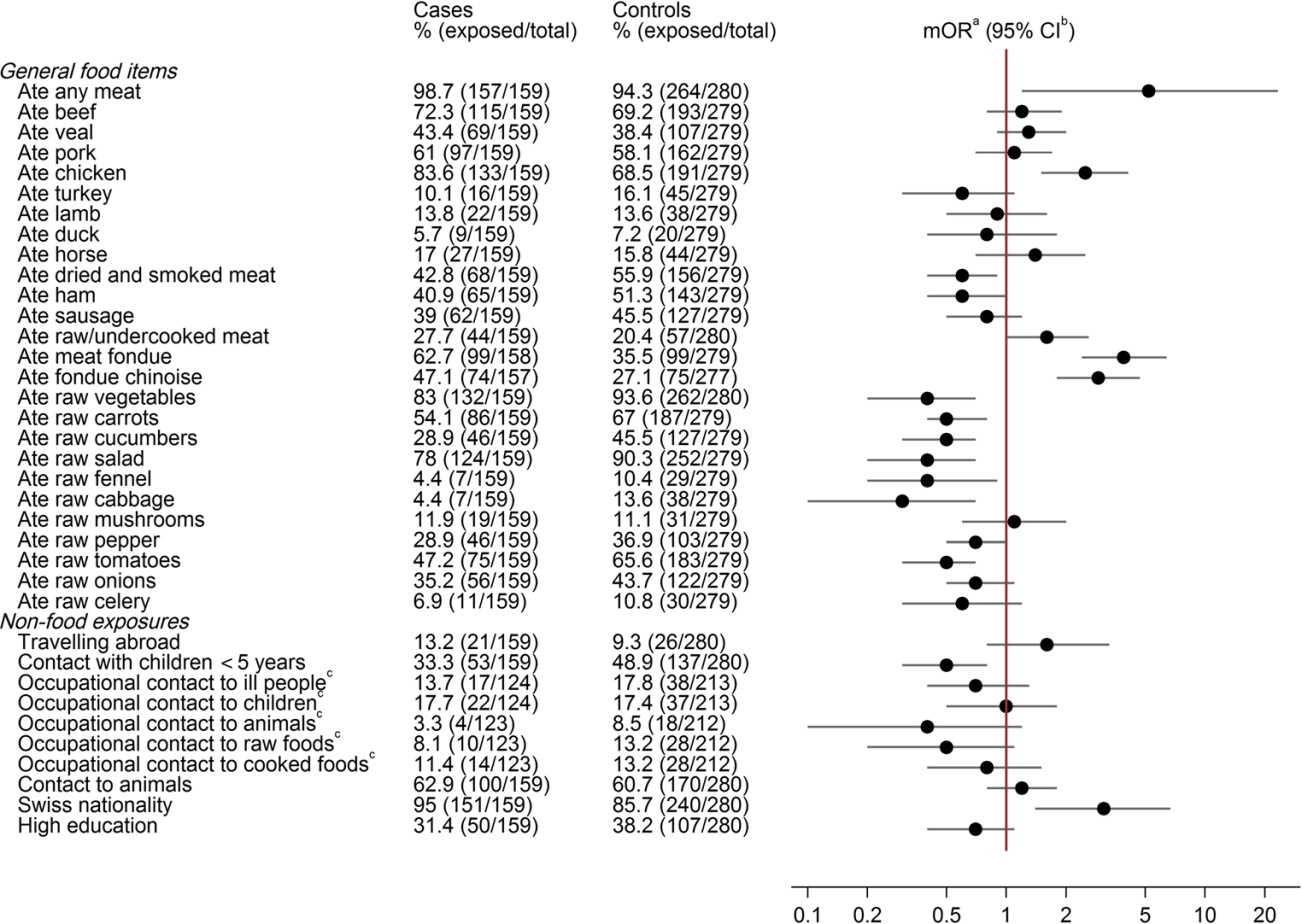
Univariable conditional logistic regression analysis of selected risk factors for campylobacteriosis in winter times (December 2012–February 2013) in Switzerland. ^a^ matched odds ratio, ^b^ confidence interval, ^c^ participants aged ≤ 15 or ≥ 65 years were excluded

Among foods consumed during the week prior to disease onset, meat consumption was identified as significant risk factor (mOR 5.2, 95 % confidence interval [CI] 1.2–23.3), but the only type of meat significantly associated with an increased risk was chicken (mOR 2.5 95 % CI 1.5–4.1) (Fig. 2). Eating raw or undercooked meat was associated with increased risk of disease (mOR 1.6, 95 % CI 1.0–2.6); however the effect was not statistically significant. Conversely, the consumption of raw vegetables was significantly associated with a decreased risk (mOR 0.4, 95 % CI 0.2–0.7). In addition, the consumption of dried and smoked meat (mOR 0.6, 95 % CI 0.4–0.9) and the consumption of ham (mOR 0.6, 95 % CI 0.4–1.0) were associated with a decreased risk.

The consumption of meat fondue was identified as a strong risk factor for disease (mOR 3.9, 95 % CI 2.4–6.4). The most frequently consumed meat fondue variant, the so-called “*Fondue chinoise*”, was also strongly associated (mOR 2.9, 95 % CI 1.8–4.7).

The univariable analysis showed no significant association of travelling abroad (mOR 1.7, 95 % CI 0.8–3.4) and campylobacteriosis. Having contact with children <5 was significantly associated with a decreased risk of illness (mOR 0.5, 95 % CI 0.3–0.8). No significant association of the disease with occupational contacts involving ill persons, animals and children, raw and cooked foods was found. The same observation was made for non-occupational contacts to animals. Swiss nationality was associated with a significantly increased risk of disease (mOR 3.1, 95 % CI 1.4–6.7). People with high education were less likely to suffer from disease (mOR 0.7, 95 % CI 0.4–1.1).

**Fig. 3:**
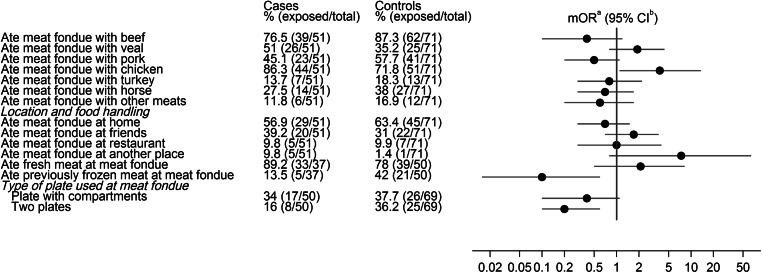
Univariable conditional logistic regression analysis of selected risk factors for campylobacteriosis related to the consumption of meat fondue in winter times (December 2012–February 2013) in Switzerland. ^a^ matched odds ratio, ^b^ confidence interval

Among the fondue consumers, chicken showed again the strongest effect (mOR 3.8, 95 % CI 1.1–13.5) of all meat types (Fig. 3). There was no noteworthy difference between fondue meals consumed at home, or outside home at friends or at restaurants. Five out of six participants who reported fondue consumption at other locations (e.g. at holiday or alpine huts) were cases. The consumption of previously frozen meat at a meat fondue was significantly associated with a decreased risk of disease (mOR 0.1, 95 % CI 0.0–0.6). The type of plate used for raw and cooked meat at a meat fondue was significantly associated with campylobacteriosis: both, using one plate with compartments and using two separate plates were associated with a decreased risk of disease (plate with compartments: mOR 0.4, 95 % CI 0.1–1.1; two plates: mOR 0.2, 95 % CI 0.1–0.6).

#### Multivariable conditional logistic regression analysis

**Fig. 4:**
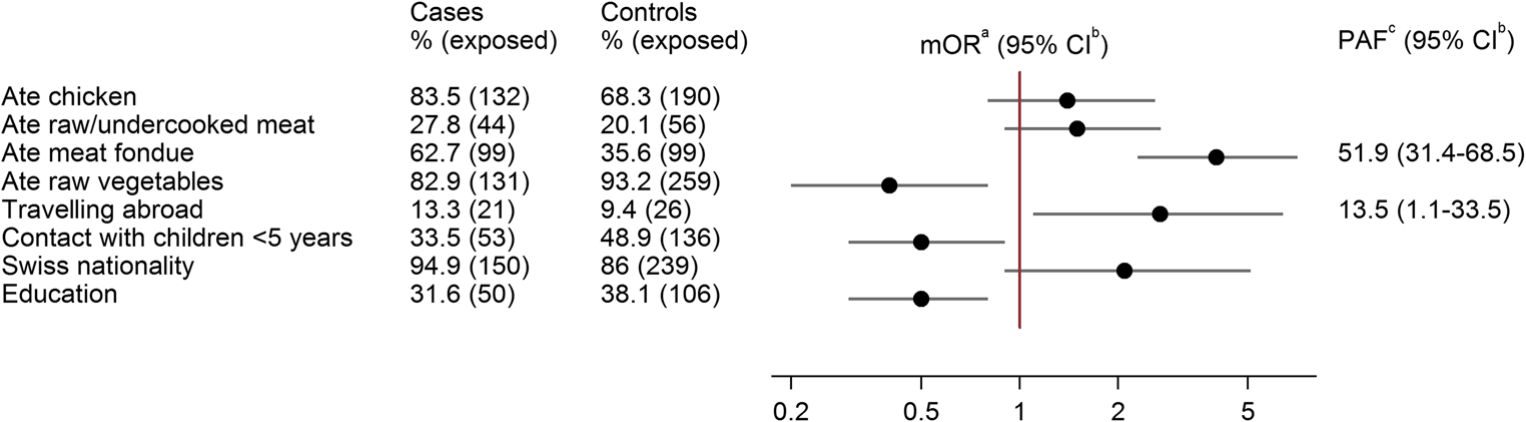
Matched multivariable conditional logistic regression analysis of selected risk factors for 158 campylobacteriosis cases and 278 controls in winter times (December 2012–February 2013) in Switzerland. ^a^ matched odds ratio, ^b^ confidence interval, ^c^ population attributable fraction

While the mOR for meat fondue remained unchanged, the effect was lower for chicken consumption in general (mOR 1.4 vs. 2.5) and for Swiss nationality (mOR 2.1 vs. 3.1) (Fig. 4). In contrast, the observed association with travelling abroad was stronger (mOR 2.7 vs. 1.7). The estimated PAFs for the significant risk factors of the multivariable model were 51.9 % (95 % CI 31.4–68.5 %) for meat fondue and 13.5 % (95 %-CI 1.1–33.5 %) for travelling abroad.

### Exploratory subgroup and stratified analyses

The stratified analysis by sex revealed a significant difference in odds for the consumption of chicken meat between females (crude OR [cOR] 4.9, 95 % CI 2.0–13.6) and males (cOR 1.4, 95 % CI 0.7–2.9). Likewise, the consumption of meat fondue increased the odds for disease among females (cOR 5.6, 95 % CI 2.9–10.8) significantly more compared to males (cOR 1.8, 95 % CI 1.0–3.3). Out of 26 cases who did not eat chicken six reported the consumption of raw or undercooked meat (23 % in cases vs. 18 % in controls), six reported meat fondue consumption with other meat types (23 vs. 15 %) but only a single person (case vs. 10 controls) reported travels abroad.

### Campylobacteriosis case characterisation

**Fig. 5:**
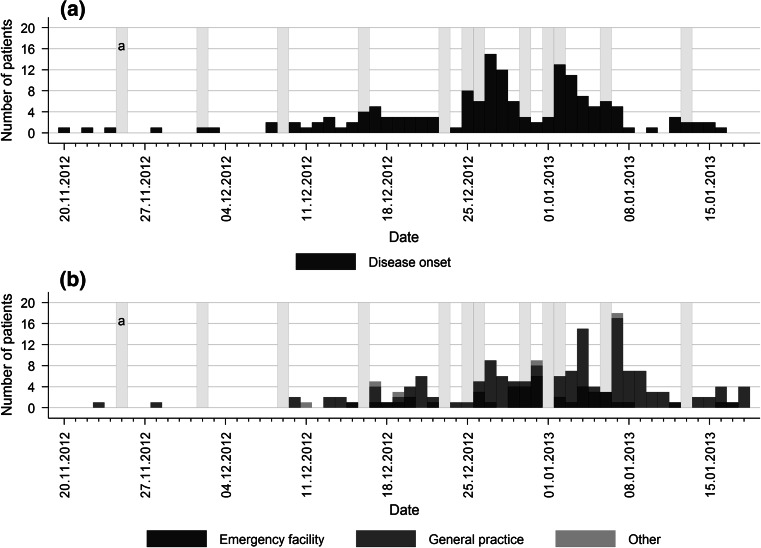
**a** Daily numbers of reported disease onsets of campylobacteriosis patients and **b** dates of consultations with a physician at an emergency facility or a general practice. ^a^ Sunday/public holiday

Most frequently reported disease onset dates were December 27th/28th and January 2nd/3rd (Fig. 5). Median duration of illness was 7 days (range 2.5–33). Only half of all patients (48 %) reported full recovery. Most commonly reported signs and symptoms were diarrhoea, abdominal pain, fever, nausea, vomiting and headache (Table 2). Other reported symptoms included limb pain, shivering, fatigue, loss of appetite and vertigo. Irrespective of their sex, more than half of the patients rated the severity of their illness as ‘severe’ denoted by a median severity score of eight on a one-to-ten scale.

**Table 2:** Campylobacteriosis in Switzerland: reported duration of illness, signs and symptoms, perceived severity, medical treatment and medication, December 2012–February 2013

	n (%) or median (range) (N = 159)
*Campylobacter*-*associated morbidity*	
Duration of illness (days)^a^	7 (2.5–33)
No recovery by the time of the interview	43 (27.0)
Perceived severity of illness^b^	8 (2–10)
*Symptoms* ^c^	
Diarrhoea	156 (98.1)
Abdominal pain	128 (80.5)
Fever	105 (66.0)
Nausea	70 (44.0)
Vomiting	54 (34.0)
Headache	20 (12.6)
*Help seeking behaviour*	
Health care seeking before consulting a physician^c^	
None: immediate consultation of a physician	52 (32.7)
Pharmacy	31 (19.5)
Medical hotline	8 (5.0)
Friends and family	68 (42.8)
Internet	23 (14.5)
Health guide	8 (5.0)
Other	10 (6.3)
Medical care seeking	
General practitioner (GP)	100 (62.9)
Emergency department	23 (14.5)
Emergency practice	19 (11.9)
Paediatrician	6 (3.8)
Medical specialist	4 (2.5)
Other	7 (4.4)
Reasons for medical care seeking^c^	
Severe symptoms	105 (66.0)
No amelioration	70 (44.0)
Need of a medical certificate	6 (3.8)
Other	44 (27.7)
*Hospitalisation*	
Total	23 (14.5)
Males^d^	13 (15.9)
Females^e^	10 (13.0)
Number of nights in hospital	3 (1–13)
*Medication*	
Consumed drugs	158 (99.4)
Drug classes^c^	
Antibiotic (Fluoroquinolones, Macrolides)	98 (61.6)
Antidiarrhoeal (Loperamide, Charcoal)	84 (52.8)
Probiotic (enterococci, saccharomyces)	73 (45.9)
Analgesic (Acetaminophen, Dipyrone, NSAIDs)	66 (41.5)
Antiemetic (Domperidone, Metoclopramide, Meclozine)	17 (10.7)
Spasmolytics (Butylscopolamine)	17 (10.7)
Acid blockers (Proton pump inhibitors)	5 (3.1)
Parenteral rehydration and/or drug application	38 (23.9)

^a^Only those recovered at time of interview included (n = 116)

^b^N = 158

^c^Multiple answers possible

^d^N = 82

^e^N = 77

#### First health care seeking

Pharmacies and medical hotlines were consulted by 20 and 5 % of the patients before seeing a physician, respectively. One third (33 %) of all patients had approached a physician directly. More than half (54 %) visited a physician within 3 days after symptoms onset. Most patients (63 %) visited a general practitioner (Fig. 5; Table 2). Emergency facilities were visited by 26 % of patients.

#### Hospitalisation

The hospitalisation rate was 14 % and did not differ between sexes, and was increased among patients ≥60 years (33 %). Half of the hospitalisations lasted at least 3 nights.

#### Pharmacotherapy

With one exception, all patients reported drug treatment; about two-thirds received antibiotics. Other medications were applied for symptomatic treatment. Among the 24 % of all patients who received an infusion for rehydration or intravenous drug application, 42 % were in outpatient treatment.

## Discussion

We assessed determinants for *Campylobacter* infections in wintertime in Switzerland with a case–control study design among laboratory-confirmed campylobacteriosis patients. A traditional meal (meat fondue), typically consumed at festive occasions in wintertime, was identified as the most important risk factor, especially if chicken meat was served. Furthermore, our findings suggest that the campylobacteriosis cases registered in the national disease registry are severely ill. The last investigation of determinants of campylobacteriosis in Switzerland dates back more than two decades and did not include the winter festive season [24].

### Factors associated with increased risk of *Campylobacter* infections

Meat fondues, predominantly “*Fondue chinoise*”, are consumed traditionally in Switzerland during dinners around Christmas and New Year. In our study, disease onset dates peaked 2–3 days after those events. This is in line with the incubation period of 2–5 days [4]. More than 50 % of *Campylobacter*-related gastroenteritis can be attributed to the consumption of meat fondue during the study period. The “*Fondue chinoise*” comprises sliced raw meat being individually handled and boiled in a family-shared broth hotpot. In contrast to chicken none of the other meat types consumed during fondue dishes were associated with *Campylobacter* infections. This is coherent with other studies identifying chicken as a risk exposure [11, 24–30]. This includes two outbreaks of *Campylobacter* infections in which meat fondue including chicken meat was the suspected source of infection [31]. Since Germans consume meat fondue with increased popularity on New Year’s Eve rather than at Christmas [32–34] *Campylobacter*-contaminated chicken could also be the cause for the peak of infections observed by Schielke et al. [23] in early January.

Further we observed that meat fondue eaters who put their raw and cooked meat on the same plate were more likely to suffer from campylobacteriosis. Conversely, the use of a compartmented plate or using two separate plates appeared to be protective in our study and has been previously recommended [35]. *Campylobacter* spp. are quickly inactivated after dipping the sliced chicken meat into the boiling broth. Therefore, on-the-plate cross-contamination of boiled meat from raw chicken meat juice is the most probable transmission route especially considering the low infectious dose of *Campylobacter* spp. [36].We found women to have significantly higher odds than men for acquiring a *Campylobacter* infection after consumption of chicken meat or meat fondue. Among our study participants women consumed more often chicken at meat fondues than men which, however, does not explain the elevated risk.

The consumption of undercooked meat as a risk factor for campylobacteriosis is well known [11, 13, 27, 28, 37]. In our study the consumption of raw or undercooked meat was associated with campylobacteriosis especially in people not consuming meat fondue. We hypothesise that the strong effect of meat fondue consumption outweighs the known effect of raw or undercooked meat consumption and, therefore, is only statistically significant in the subgroup of people not consuming meat fondue. Travelling abroad was the only behavioural factor in the multivariable analysis significantly associated with increased odds for *Campylobacter* infections. This risk factor has been described previously for Switzerland [24] and other countries [11, 25, 26, 28, 30]. Further, almost all acute gastroenteritis patients with travel history are tested for gastrointestinal pathogens and are more likely to be diagnosed (personal communication).

One can argue that meat fondue represents an intermediate variable on the pathway from chicken consumption to *Campylobacter* spp. infection. Intermediate variables, if included in the multivariable analysis, might bias the estimates—usually towards the null. Therefore, we re-ran the regression models omitting meat fondue-consumption: as expected, chicken consumption showed a higher odds ratio (2.3) compared to the full model. The point estimates for all other variables remained similar, with the exception of travelling abroad which was associated with a smaller effect.

### Factors associated with reduced risk of *Campylobacter* infections

The finding that a reduced risk of disease is associated with having contact to children <5 years is difficult to interpret; especially because a high incidence is noticed for this age-class in the NNSID [1]. Persons having contact with young children may differ in general and food hygiene and dietary habits [38]. High education was associated with a reduced risk of disease. The association with gastrointestinal diseases in high-income countries is discussed controversially [38–41]. Another factor associated with a decreased risk was the consumption of raw vegetables. Similar findings are described from several European countries and elsewhere [13, 25, 27, 28, 42] linking the protective effects of the consumption of raw vegetables to high amounts of antioxidants and carotenoids which act as bacterial growth inhibitors and generally increase immunity to infection. Several reports underscore that people who eat raw vegetables differ from others concerning cooking and eating preferences and behaviour [13, 25, 27, 28, 42]. The consumption of raw vegetables, especially during winter time, may reflect a generally healthy lifestyle [25, 27, 28, 42].

An exploratory subgroup analysis among meat fondue consumers indicates that consuming previously frozen meat is associated with a decreased risk of campylobacteriosis. Similar experiences were made in Iceland where the number of campylobacteriosis cases declined after freezing of meat originating from *Campylobacter*-infected broiler flocks [43]. In Switzerland, Baumgartner et al. [44] showed that chicken products were less contaminated with *Campylobacter* spp. after freezing,—a finding which is corroborated by the studies in Iceland [45] and Norway [46].

In summary, risk and preventive factors in this study point at contamination risks upstream at food production- and downstream at retail- and consumer sides. Consequently, potential preventive risk reduction measures could be applied upstream and downstream: upstream -, through decontamination at slaughter using peracetic acid [47] resulting in a decreased bacterial load at retail level or freezing of chicken meat before reaching retail [43, 45, 46]. Downstream risk prevention measures could include improving consumer awareness in handling raw chicken meat additionally to the current hygiene notice on Swiss chicken meat packages.

### Illness perception and treatment of acute campylobacteriosis

Patients suffering from *Campylobacter* infection reported typical symptoms of an acute gastroenteritis and a high perceived severity of illness. Comparable studies for Switzerland are lacking; however, the pattern is coherent with experiences from other countries [13, 48–51]. The reported severity of illness appears to be slightly higher compared to others [48]. Compared to other countries the proportion of hospitalised patients (14 %) was higher [13, 48] or slightly lower [52]. This variability could be due to differences in health systems, including differing notification criteria, case definitions and health care provider structures.

Although antibiotics are not generally recommended for treatment of campylobacteriosis more than 60 % of our study patients received antibiotic treatment. In absence of information on the individual patient’s medical history we cannot judge whether antibiotic use was medically indicated.

Generally, case-fatality rates in high-income countries range from 0.04 to 0.6 % [2, 52–54]. We observed no death during our study. However, due to the similarity of epidemiological patterns in Europe *Campylobacter*-attributable mortality is likely to occur also in Switzerland [2, 54].

### Strengths and limitations

We recruited all our cases from laboratory-confirmed campylobacteriosis patients registered in the NNSID. Patients with a mild course of disease are less likely to consult a physician or to be tested for campylobacteriosis and, hence, less likely to be notified. Participating laboratories were from the private sector only; therefore, the hospitalisation rate and the proportion of patients approaching emergency departments and policlinics directly may be underestimated. Similarly, recruiting cases from private laboratories, serving mainly general practitioners, could explain the imbalance in nationalities. Swiss nationals more often consult their general practitioners while non-Swiss are more likely to approach emergency departments. As expected, patients more often volunteered to participate in the study and contacted back the study team after initial contacting failed. Cases may remember their exposures more accurately than controls, since they might have been reflecting about what caused their illness. Nevertheless, “don’t know” was answered equally often by cases and controls. In addressing potential biases from recalling exposure risks we applied photo-illustrated questionnaires.

## Conclusion

The study provides strong evidence that the consumption of a national festive dish (“*Fondue chinoise*”) is a risk factor for human campylobacteriosis in Switzerland. The main risks associated with this dish are probably twofold: firstly, chicken meat is frequently contaminated with *Campylobacter* spp. [44]. Secondly, the possibilities of and occasions for cross-contamination and ingestion of bacteria are manifold and the infection risk is exacerbated through individual food-handling at the table. Our findings, therefore, highlight the importance of food hygiene for chicken preparation and consumption at meat fondues. The steadily increasing number of notified campylobacteriosis cases, the high population attributable fraction for meat fondue and the previously unknown severity of illness and hospitalisation rate underline the relative importance for Swiss public health over the festive season and point toward the necessity for public health interventions. Prevention measures could include decontamination of chicken meat at slaughter resulting in a decreased bacterial load at retail level, freezing of chicken meat before reaching retail and improving consumer awareness in handling raw chicken meat.
